# Supplementary figures and images for: Limited differential expression of miRNAs and other small RNAs in LPS-stimulated human monocytes
Source: PLoS One. 2019 Mar 25;14(3):e0214296. doi: 10.1371/journal.pone.0214296 (PMC6433273; doi:10.1371/journal.pone.0214296)

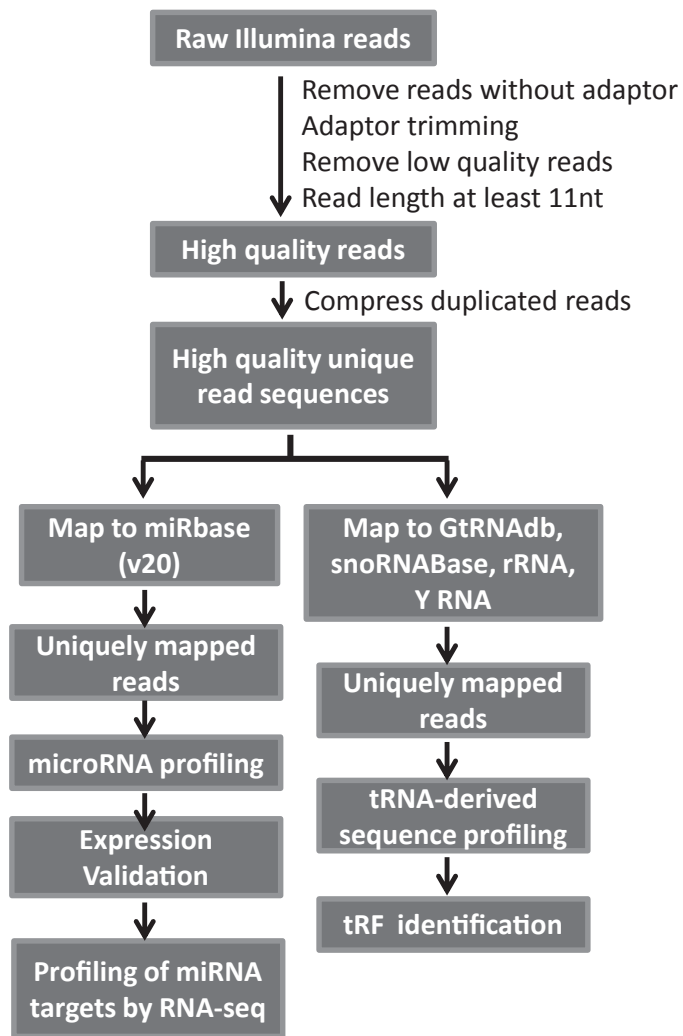

Supplementary Figure 1

Supplement: S1 Fig — Low quality sequencing reads were first filtered by removing low quality reads (Phred score<30 or no sequencing adaptor), removing read fragments less than 12 nucleotides in length, and trimming adaptor sequences flanking the reads. Unique sequences were then aligned either miRbase (v20) and other small RNA reference databases that included human tRNA sequences from GtRNAdb, snoRNA/scaRNA sequences from snoRNABase, as well as ribosomal RNAs and hY RNAs 1/3/4/5 from NCBI, using OmicSoft Aligner (V5.1). Multi-mapped reads were discarded while uniquely mapped reads were retained for downstream small RNA identification, profiling, and validation. (PDF) [file pone.0214296.s001.pdf]

A

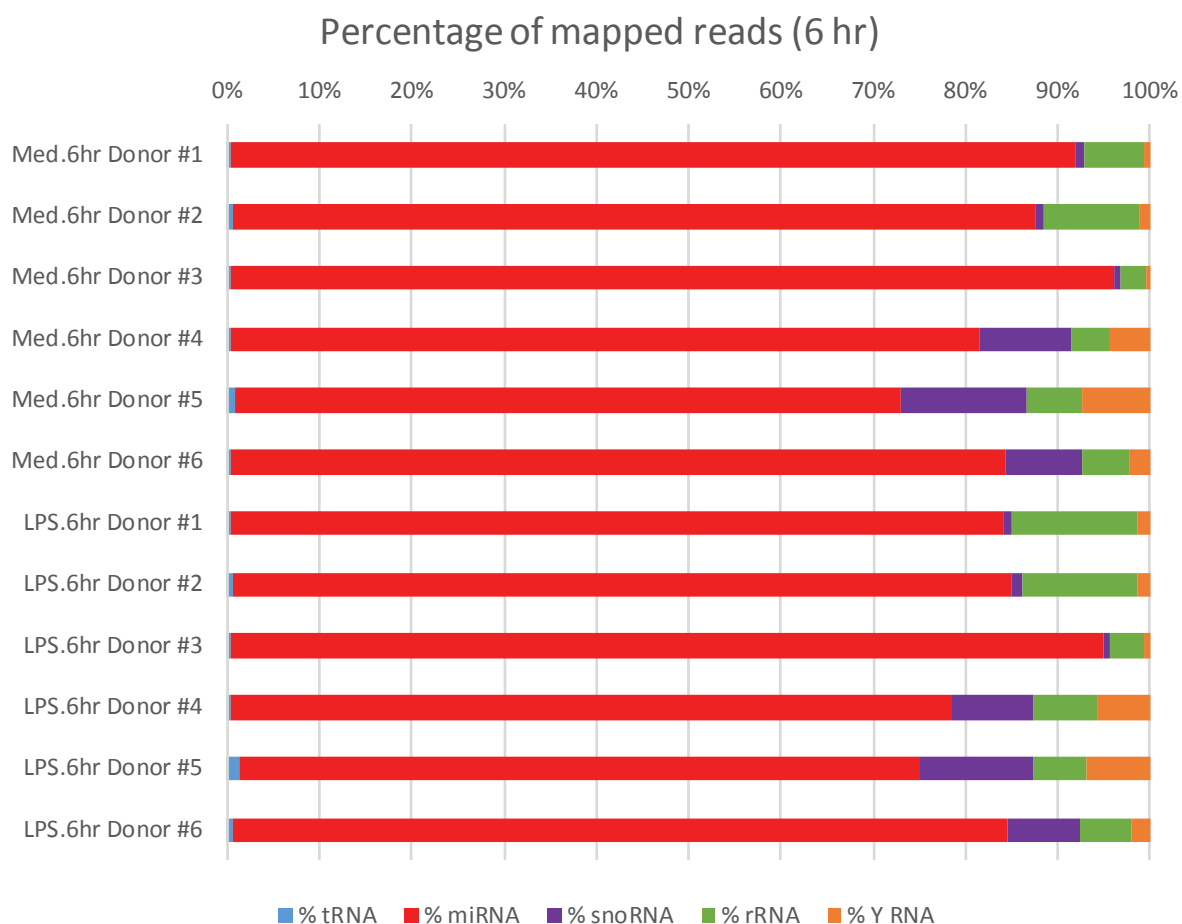

B

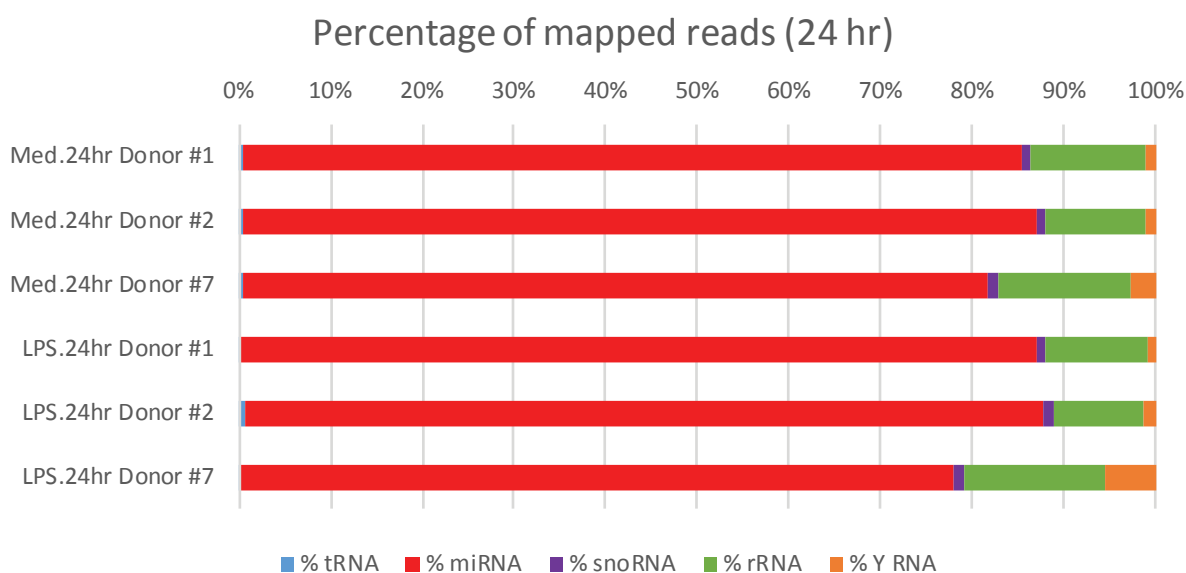

Supplement: S2 Fig — The percentage of reads aligned to the various RNA species are indicated in the bar plots. Blue = tRNA, red = miRNA, purple = snoRNA, green = rRNA, orange = Y-RNA. (A) shows samples sequenced after 6 hours of treatment, while (B) shows samples sequenced after 24 hours of treatment. For all samples, 72.1–95.7% of reads aligned to miRNAs. (PDF) [file pone.0214296.s002.pdf]

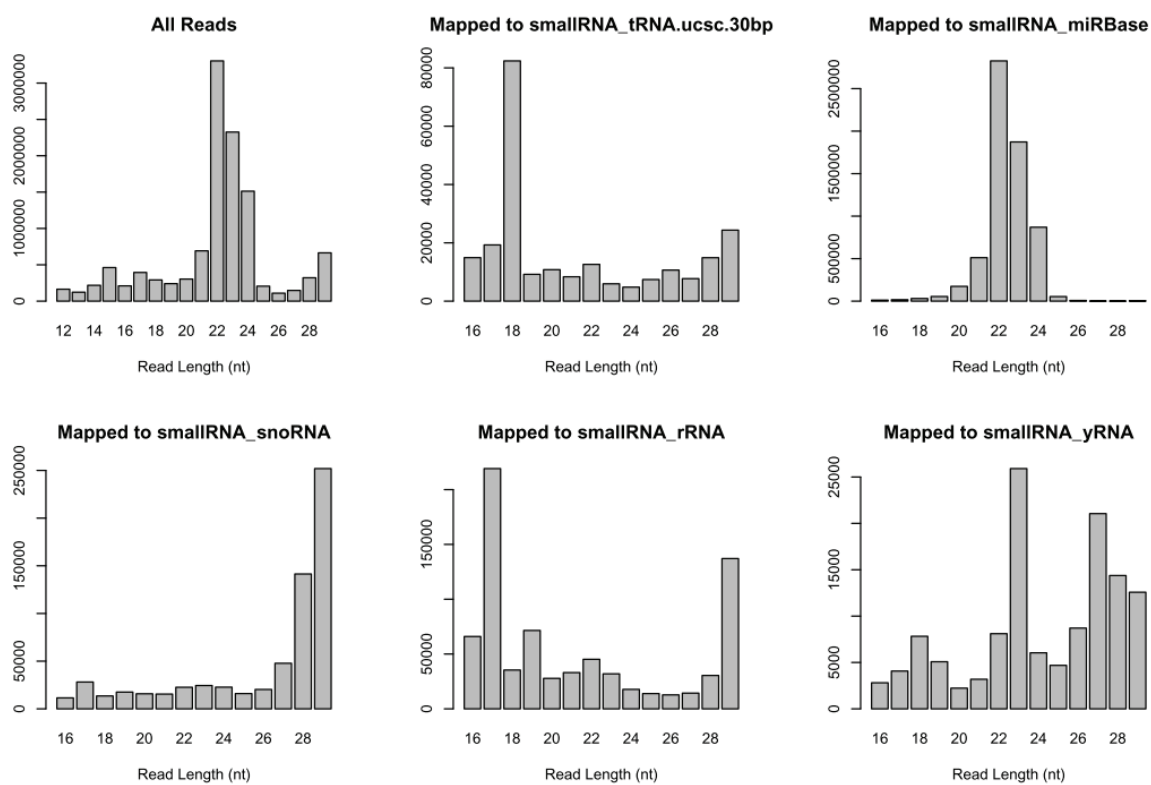

Supplementary Figure 3

Supplement: S3 Fig — The distribution of the insert sizes of the small RNA libraries show that the insert sizes of the reads were predominantly 19bp to 24bp in length, whereas the length of the inserts for miRNA ranged from 20 to 24bp and that of tRNA-derived small RNAs was concentrated at 18bp. (PDF) [file pone.0214296.s003.pdf]

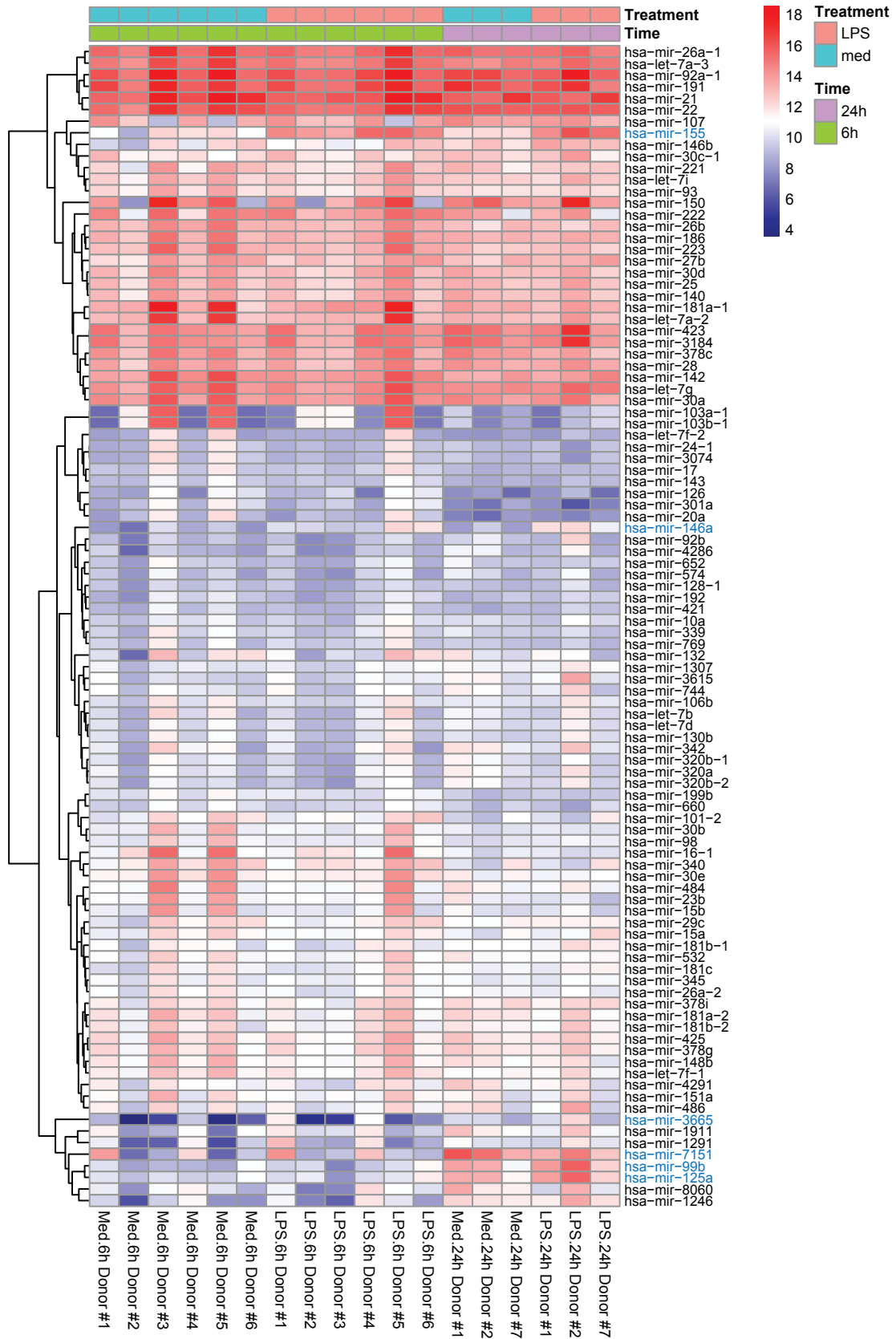

Supplementary Figure 4

Supplement: S4 Fig — Normalized expression of miRNAs is shown in log2(counts per million + 1) for each sample. Blue miRNAs denote significantly miRNAs at either 6 hours or 24 hours post-stimulation with LPS. (PDF) [file pone.0214296.s004.pdf]

A

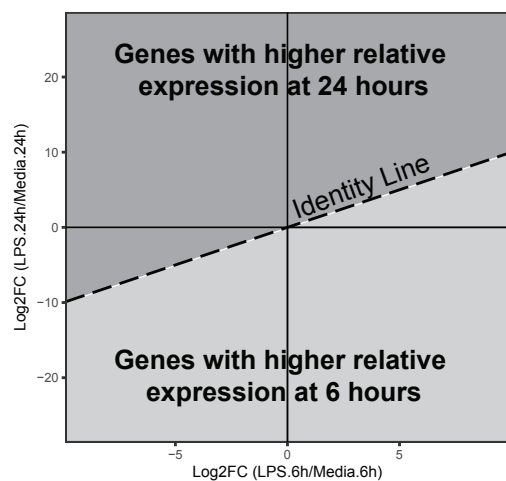

B

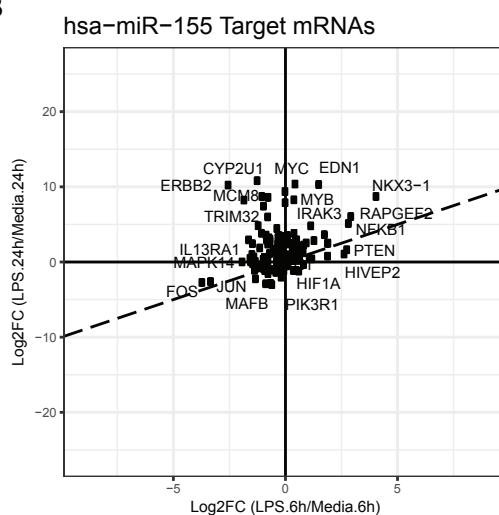

C

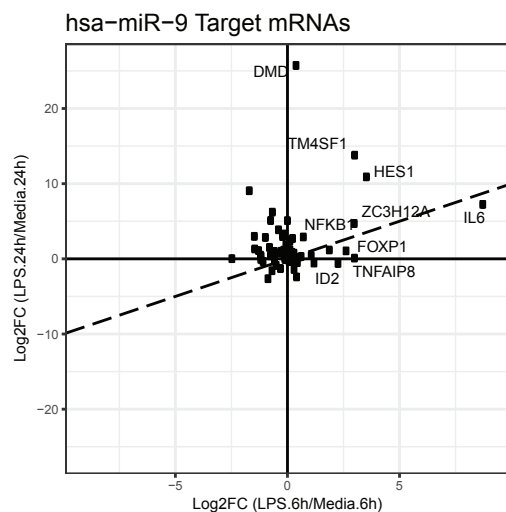

D

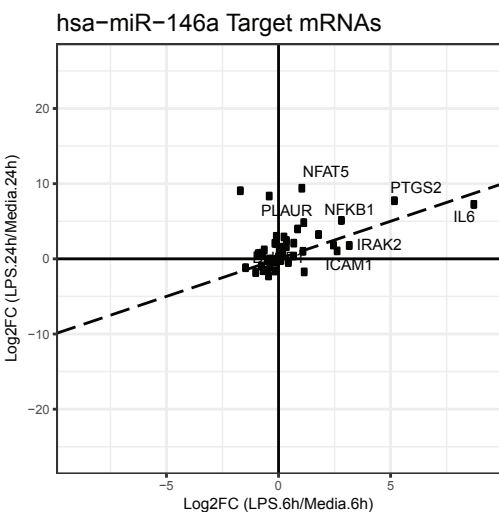

E

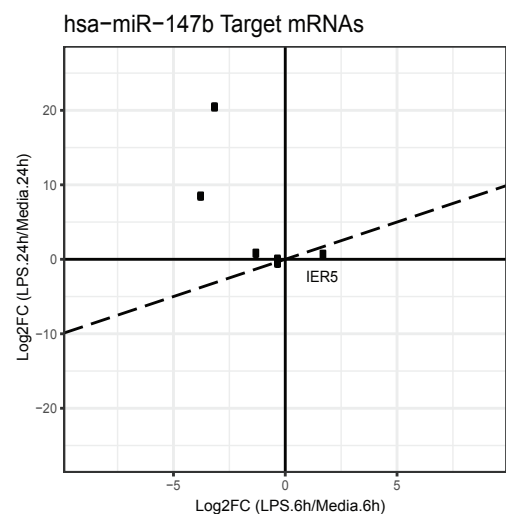

F

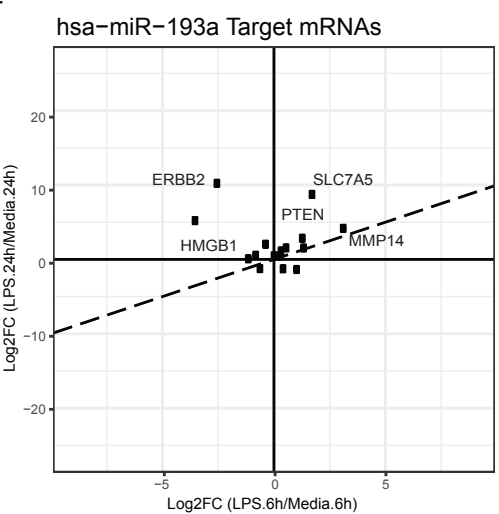

Supplement: S6 Fig — (A) Diagram depicting the log-fold change expression of miRNA-regulated genes after 6 hours and 24 hours of LPS stimulation. (B-E) Log-fold change RNA-seq gene expression at 6 and 24 hours of previously validated targets of miRNAs hsa-mir-155 (B), hsa-mir-9 (C), hsa-mir-146a (D), and hsa-mir-147b (E), and hsa-mir-193a (F). Black dots represent individual genes that are targets of a specific miRNA. Significantly differentially expressed genes are labeled. Dotted line represents the unity line. (PDF) [file pone.0214296.s006.pdf]

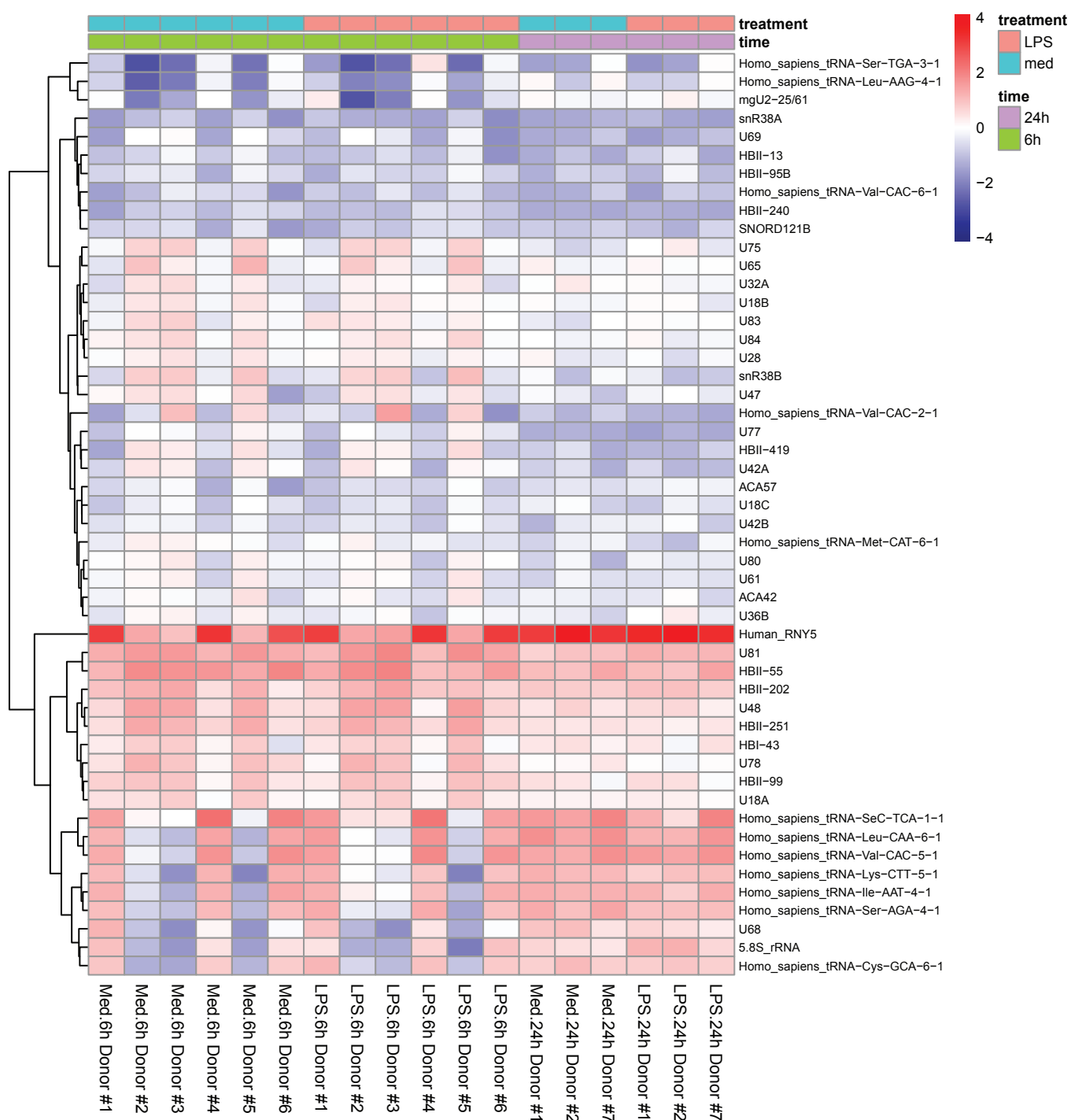

Supplementary Figure 7

Supplement: S7 Fig — Z-score expression heatmap of the top 50 most variant non-miRNA small RNAs in LPS-stimulated monocytes after 6 hours and 24 hours of treatment. No small RNAs had significant differential expression. (PDF) [file pone.0214296.s007.pdf]

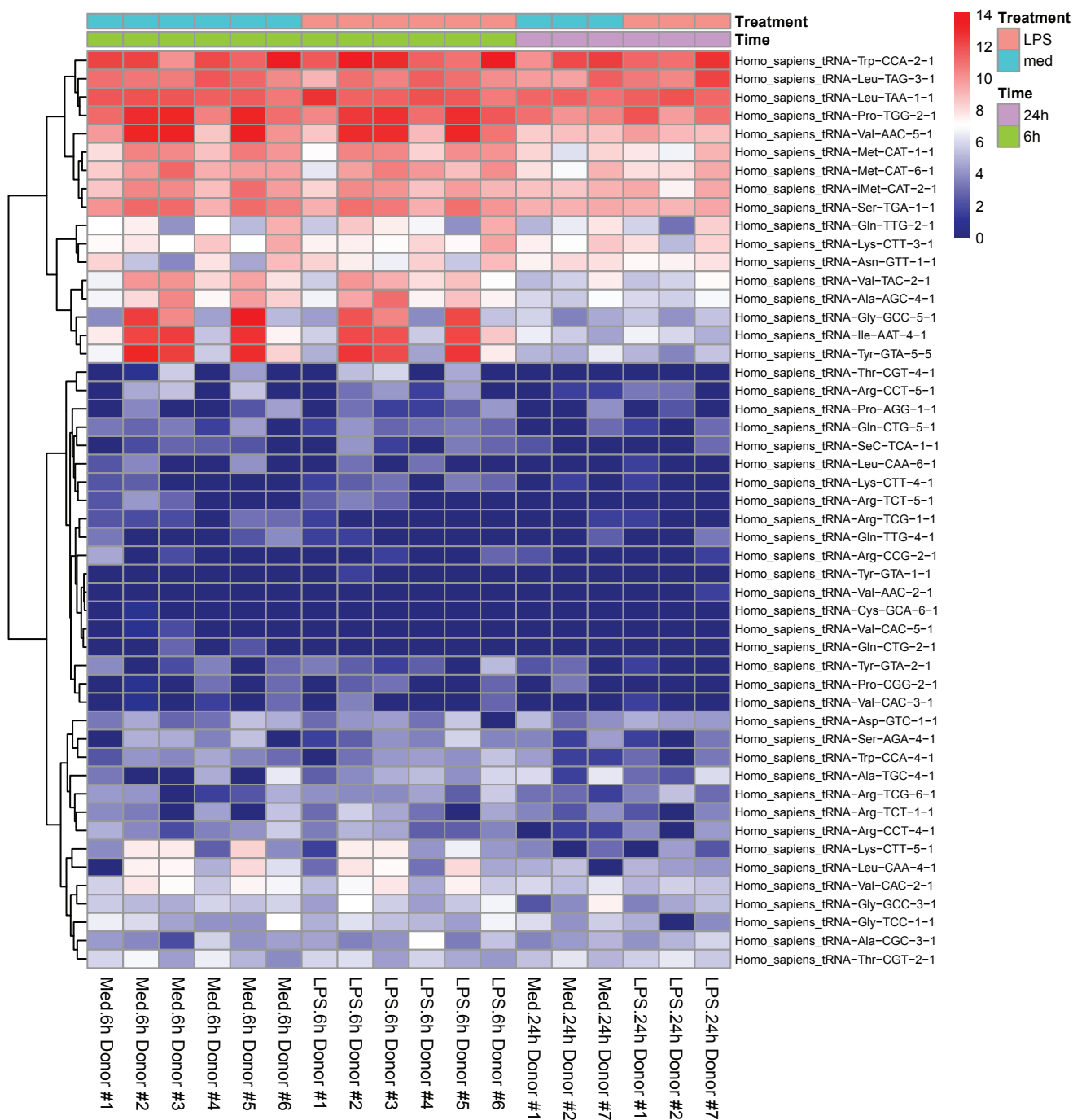

Supplementary Figure 8

Supplement: S8 Fig — Log2-fold expression heatmap of the top 50 tRNAs with the highest average small RNA-seq expression across all samples. (PDF) [file pone.0214296.s008.pdf]
