# Supplementary material for: Limited differential expression of miRNAs and other small RNAs in LPS-stimulated human monocytes
Source: PLoS One. 2019 Mar 25;14(3):e0214296. doi: 10.1371/journal.pone.0214296 (PMC6433273; doi:10.1371/journal.pone.0214296)
Supplement: S5 Fig — The top ten upregulated/downregulated GO terms with the lowest q-values are shown. Dot size represents the number of differentially expressed genes present for each GO term. Dot color represents the FDR-adjusted q-value (FDR = 0.01) for each GO term. (PDF) [file pone.0214296.s005.pdf]

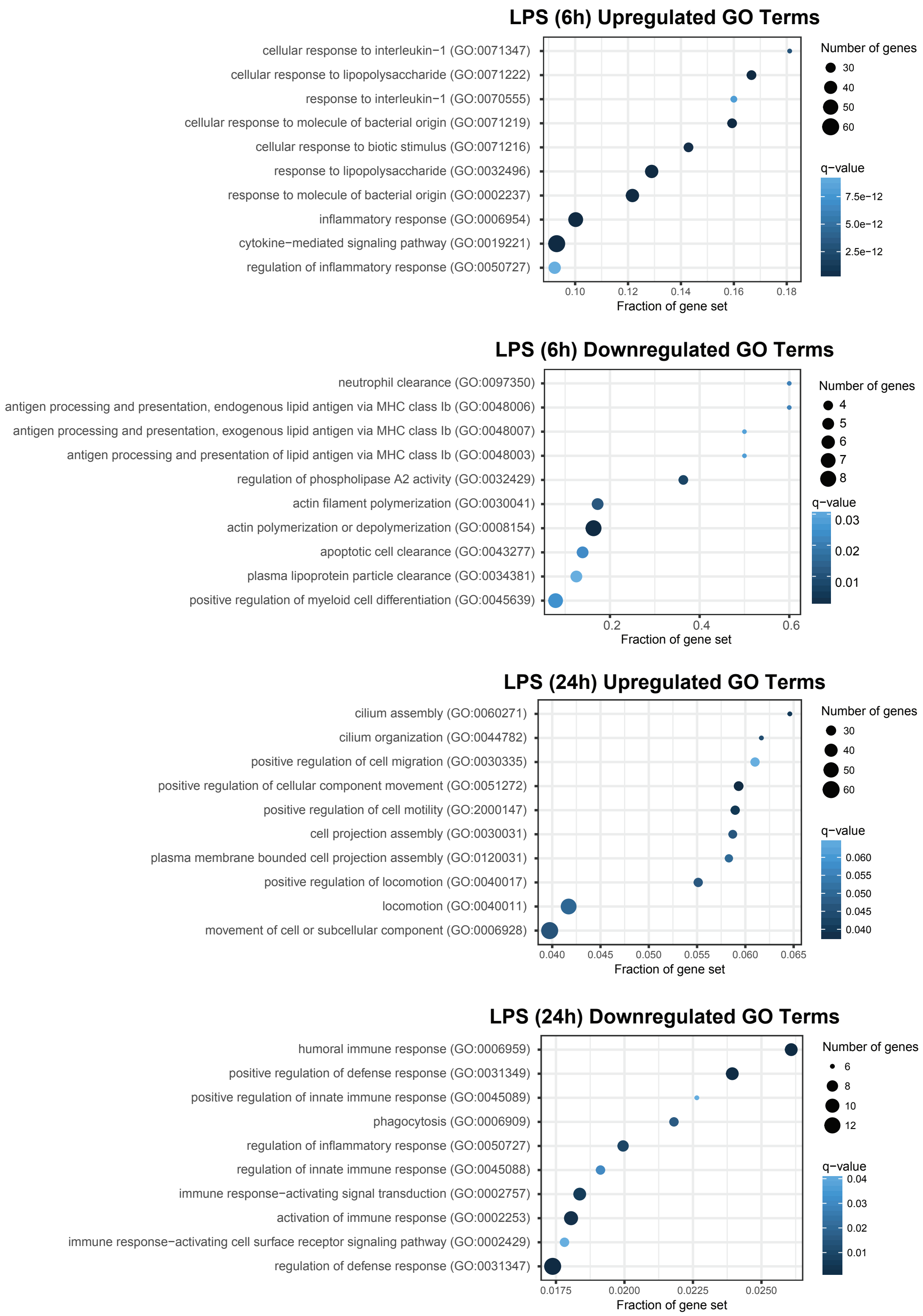

Supplementary Figure 5
